# Prevalence and associated factors of unilateral spatial neglect among patients with stroke at Mbarara Regional Referral Hospital, Uganda: a cross-sectional study

**DOI:** 10.21203/rs.3.rs-6778985/v1

**Published:** 2026-01-04

**Authors:** Asiimwe Derrick, Migisha Richard, Nuwahereza Amon, Nkoyooyo Dauglaus, Lubwama Conrad, Namayanja Rosemary, Omolo Ouma Ronald, Martha Sajatovic, Josephine Najjuma, Mark Kaddumukasa, Elly Katabira, Kira Bullock, Agaba David Collin

**Affiliations:** 1 Department of Physiology, Mbarara University of Science and Technology, Mbarara, Uganda; 2 Department of Physiotherapy, Mbarara Regional Referral Hospital, Mbarara, Uganda; 3 Department of Psychiatry and Neurology, Case Western Reserve University School of Medicine, Cleveland, Ohio, USA; 4 Department of Nursing, Mbarara University of Science and Technology, Mbarara, Uganda; 5 Department of Medicine, Makerere University College of Health Sciences, Kampala, Uganda; 6 Department of Neurology, Duke Global Neurosurgery & Neurology, Duke University, Durham, North Carolina, USA

**Keywords:** Unilateral spatial neglect, stroke, prevalence, cerebrovascular disorders, Stroke rehabilitation, Uganda

## Abstract

**Background:**

Unilateral spatial neglect (USN) is a common neurocognitive deficit following stroke. It impairs rehabilitation and functional outcomes, particularly in low-resource settings where data are limited. This study determined the prevalence of USN and its associated factors among patients with stroke at Mbarara Regional Referral Hospital (MRRH), southwestern Uganda.

**Methods:**

We conducted a cross-sectional study from August 12 to December 15, 2024. Patients with a recent stroke event within the preceding 0–4 months receiving care at MRRH were enrolled using consecutive sampling. Patients who were unable to see or were aged <18 years were excluded. USN was assessed using the Sunnybrook Neglect Assessment Procedure (SNAP): scores >5 indicating USN. Sociodemographic, clinical, and lifestyle factors were collected via structured questionnaires. Multivariable modified Poisson regression was used to identify factors associated with USN, reporting adjusted prevalence ratios (aPR) with 95% confidence intervals (CI).

**Results:**

We enrolled 117 participants with a median age of 68 years (interquartile range [IQR]: 56–78). Most were female (65.8%). The prevalence of USN was 53.8% (n=63; 95% CI: 44–63). USN was more prevalent in the acute phase (60%) compared to the subacute (32%) and chronic phases (8%). Factors significantly associated with USN included severe stroke (aPR=1.47, 95% CI: 1.12–1.92, p=0.005), right-hemisphere lesions (aPR=1.58, 95% CI: 1.14–2.19, p=0.006), age ≥60 years (aPR=1.66, 95% CI: 1.02–2.72, p=0.042), and right-handedness (aPR=0.94, 95% CI: 0.94–0.99, p<0.001). \

**Conclusion:**

This study revealed a high prevalence of USN among patients with stroke at a referral hospital in southwestern Uganda. Stroke severity, right-hemisphere lesions, advanced age, and right-handedness were associated with USN. Targeted rehabilitation and early screening for patients with stroke with risk factors, particularly those with severe strokes or advanced age, could optimize recovery and improve long-term outcomes.

## Introduction

Stroke is a major global health burden, contributing significantly to disability and mortality, particularly in low- and middle-income countries (LMICs) where healthcare resources are limited(1). In sub-Saharan Africa, the incidence of stroke has risen due to increasing cardiovascular risk factors such as hypertension and diabetes, with Uganda reporting a stroke incidence of approximately 145 per 100,000 people annually(2). Unilateral spatial neglect (USN), a neurocognitive deficit characterized by the failure to attend to stimuli on one side of the body or environment, affects 20–85% of stroke survivors, severely impacting rehabilitation, mobility, and quality of life(3). USN not only prolongs hospital stays but also increases the risk of falls and dependency, posing significant challenges to functional recovery(4). USN is often associated with right-hemisphere lesions, leading to neglect of the contralesional side, and is exacerbated by factors such as stroke severity, age, and lesion size (5).

In LMIC settings, where rehabilitation services are often inadequate, the burden of USN is likely amplified, yet data on its prevalence and associated factors remain scarce (6). In Uganda, stroke is a leading cause of morbidity (2), with Mbarara Regional Referral Hospital (MRRH), a tertiary care center in southwestern Uganda, managing approximately 25 patients with stroke monthly. Despite this high burden, there is a lack of routine USN screening at MRRH, hindering targeted interventions. Routine USN screening using feasible tools such as Sunnybrook Neglect Assessment Procedure (SNAP) is crucial for identifying patients at risk of prolonged recovery, increased falls, and dependency, enabling early, tailored rehabilitation to improve functional outcomes and reduce healthcare costs. This study aimed to determine the prevalence of USN and its associated factors among patients with stroke at MRRH, providing evidence to inform clinical practice and improve stroke care in resource-constrained settings.

## Methods

### Study design and setting

This cross-sectional study was conducted from August 12 to December 15, 2024, at MRRH, a 500-bed tertiary hospital in Mbarara City, southwestern Uganda, located along the Mbarara-Kabale Highway (coordinates: 0.6164S, 30.6589E). MRRH serves as a major referral center for southwestern Uganda, managing patients with stroke at emergency, medical, neurology, and physiotherapy units in the catchment area of Mbarara city, Isingiro, Bushenyi, Sheema, Ibanda, Kazo, Buhweju, Mitooma, Rubirizi, and Kiruhura districts. MRRH provides stroke care through its emergency unit, medical wards, neurology clinic, and physiotherapy department. Patients presenting with acute stroke are initially evaluated and stabilized in the emergency care unit before admission to the medical ward for further management. Diagnostic services for stroke include clinical evaluation supported by neuroimaging, primarily computed tomography (CT) scanning which is mandatory for all patients with stroke. At the time of the study, advanced acute stroke interventions such as intravenous thrombolysis and endovascular thrombectomy were not routinely available. There is no dedicated stroke unit; however, stroke care is delivered through a multidisciplinary team approach involving physicians, medical officers, nurses, physiotherapists, and other allied health professionals. On average, approximately 25 stroke patients are admitted to MRRH per month, and patients typically receive rehabilitative care for a period of 3 to 6 months depending on clinical needs and functional recovery.

### Study population, sample size, and sampling

We enrolled patients with stroke within 0–4 months of stroke onset, using consecutive sampling from both inpatient and outpatient services. Inpatients were recruited from the emergency care unit and medical wards, while outpatients were recruited from the neurology and physiotherapy outpatient clinics. Inclusion criteria included a CT scan-confirmed stroke diagnosis by a physician and a CT-scan is completed by more than 95% of the admitted patients. Exclusion criteria were inability to see (due to limitations of the assessment tool), age <18 years (as the tool is not validated for pediatric populations) and having bilateral strokes

Sample size was determined based on an expected USN prevalence of 50% (conservative estimate), using the Kish Leslie formula with a 95% confidence level (Z=1.96) and a 5% margin of error, yielding a minimum sample of 106. Adjusting for a 10% non-response rate, the final sample size was 117 participants.

### Data collection and study variables

Unilateral spatial neglect (USN) was assessed using the SNAP, a standardized paper-and-pencil screening tool comprising line bisection, star cancellation, line crossing, and figure copying tasks(4). SNAP scores range from 0 to 100, with scores greater than 5 indicating the presence of unilateral spatial neglect. The SNAP has demonstrated strong reliability, with high inter-rater agreement (92%) and excellent test-retest reliability (r=0.95), supporting its consistency for repeated assessment of USN(4). In validation studies comparing SNAP with the Visual Scanning Behavior (VSB) task, the tool demonstrated an overall sensitivity of 68% and specificity of 76% for detecting unilateral spatial neglect. Among SNAP subtests, the shape cancellation task showed the highest sensitivity (70%), while the drawing/copying task demonstrated the highest specificity (99%) (4). Administration of the SNAP takes approximately 10–15 minutes and requires minimal training, allowing its use by both clinicians and trained non-clinician research assistants in resource-limited settings. Stroke severity was measured with a standardized National Institutes of Health Stroke Scale (NIHSS), categorized as mild (0–4), moderate (5–15), or severe (16–42)(7). Stroke phase was categorized using commonly accepted clinical definitions based on time since stroke onset. Acute stroke was defined as ≤7 days post-stroke, subacute stroke as >7 days to <6 months, and chronic stroke as ≥6 months post-stroke. Pre-stroke functional status was evaluated using the modified Rankin Scale (mRS) adopted and categorized as independent (0–2) or dependent (3–5) (8). Sociodemographic factors (age, sex, education, occupation) and clinical factors included stroke type (ischemic or hemorrhagic), lesion location (right or left hemisphere), and handedness (right or left-handed). Comorbidities assessed included hypertension. Age was categorized as <60 and ≥60 years to distinguish younger and older participants, based on prior literature demonstrating clinically meaningful differences in stroke characteristics, vascular risk profiles, and outcomes at this threshold (22,23) The data were collected via structured questionnaires administered by trained research assistants.

Research assistants were trained on SNAP administration, questionnaire delivery, and ethical considerations. The study tools were pretested on 12 patients with stroke at Mayanja Memorial Hospital to assess clarity, feasibility, and consistency of administration prior to the main data collection. Ability to sustain attention and follow instructions was assessed clinically during SNAP administration, and participants unable to complete the assessment due to cognitive, attentional, or language impairments were excluded. USN was defined as a SNAP score >5, based on established cutoffs for detecting neglect (3). Patients were classified as having USN (SNAP score >5) or not having USN (SNAP score ≤5).

### Data management and analysis

Data were entered into Kobo Collect and exported to Stata version 17 (Stata Corp, College Station, TX, USA) for analysis. Descriptive statistics (frequencies, median, proportions, means, and standard deviations) summarized participant characteristics. The Shapiro-Wilk test was used to test for normality or skewness, which informed us whether to use the mean or median. Comparisons between participants with unilateral spatial neglect (USN+) and those without unilateral spatial neglect (USN−) were performed using Chi-square test for categorical variables. The prevalence of USN was calculated as the proportion of participants with SNAP scores >5, and was reported with corresponding 95% confidence interval (CI). Bivariate analyses using modified Poisson regression assessed associations between USN and independent variables. Multivariable modified Poisson regression with robust standard errors was used to identify factors associated with USN, reporting adjusted prevalence ratio (aPR) with 95% CI (9). Variables with p<0.2 in bivariate analysis, along with biologically plausible factors were included in the multivariable model. Modified Poisson regression was chosen instead of logistic regression because the outcome (USN) was common (>50% prevalence), and logistic regression would overestimate effect sizes in this setting (24). The statistical significance was set at p<0.05.

## Results

**Figure 1.**
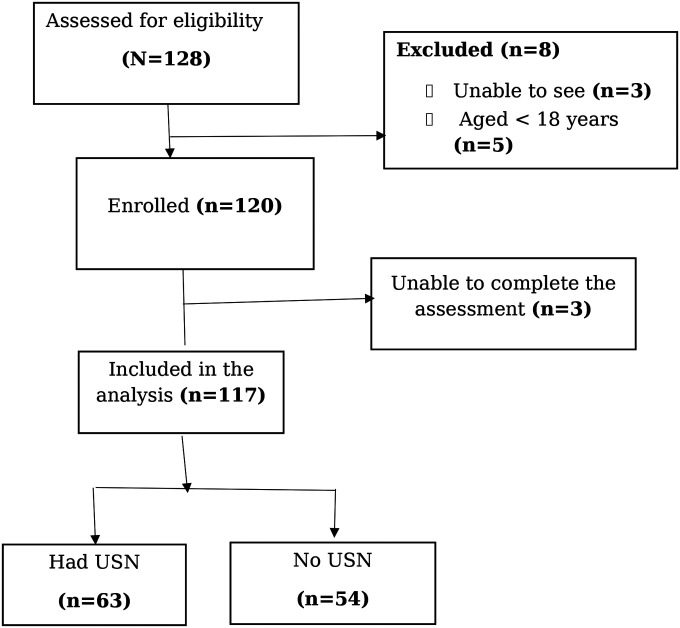
Flow diagram of eligibility, exclusions, and final analytical sample of patients with stroke Mbarara Regional Referral Hospital, Uganda, August-December 2024; USN: unilateral spatial neglect

During the study period, 128 patients with stroke were assessed for eligibility. Of these, 120 met the inclusion criteria and were recruited. Three participants were unable to complete the assessment, resulting in a final analytical sample of 117 participants (Figure 1).

### Characteristics of study participants

We enrolled 117 patients with stroke, with a median age of 68 years (IQR: 56–78). The majority were female (65.8%), had low education (80.2%), were unemployed (67.5%), and were functionally independent pre-stroke (91.4%). Clinically, most participants experienced ischemic strokes (71.8%) of moderate severity (65.8%), with right-hemisphere lesions in 53.8%, and were in the acute phase (61.5%). The majority were right-handed (89.7%), 65.8% had moderate stroke severity, 17.1% had severe stroke (Table 1).

The prevalence USN was higher among older participants, with 68.3% of participants aged ≥60 years exhibiting USN compared to 31.7% among those aged <60 years (p=0.001). Additionally, USN was more common among participants who were functionally dependent prior to stroke (12.7% vs 3.7% independent; p=0.018), those not employed (69.8% vs 30.2% employed; p=0.008), and participants with a history of hypertension (50.8% vs 37.0% without hypertension; p=0.044). No significant differences were observed between USN and non-USN participants in terms of sex, education level, stroke type, stroke phase, or handedness (Table 1).

### Prevalence of unilateral spatial neglect

Of the 117 participants, 63 had USN, for a prevalence of 53.8% (95% CI: 44–63). USN prevalence was highest in the acute phase (60%), followed by the subacute phase (32%), and lowest in the chronic phase (8%).

### Factors associated with unilateral spatial neglect

Severe strokes, right-hemisphere lesions, older age, and handedness were significantly associated with USN at multivariable analysis (Table 2). Participants with severe stroke had a 1.47 times higher prevalence of USN (aPR=1.47, 95% CI: 1.12–1.92, p=0.005) compared to those with mild stroke. Those with right-hemisphere lesions had a 1.58 times higher prevalence (aPR=1.58, 95% CI: 1.14–2.19, p=0.006) compared to left-hemisphere lesions. Participants aged ≥60 years had a 1.66 times higher prevalence (aPR=1.66, 95% CI: 1.02–2.72, p=0.042) compared to those <60 years. Right-handedness was associated with a 6% lower likelihood of experiencing USN (aPR=0.94, 95% CI: 0.94–0.99, p<0.001). Pre-stroke functional status showed a marginal association (aPR=1.19, 95% CI: 0.98–1.45, p=0.070). Other variables, including sex, stroke type, and hypertension history, were not significantly associated with USN (p>0.05) (Table 2).

## Discussion

Approximately half of the patients with stroke surveyed at the regional referral hospital, southwestern Uganda were found to have USN. USN prevalence was highest in the acute phase. Significant associations were found between USN and stroke severity, right-hemisphere lesions, advanced age, and right-handedness.

The USN prevalence of 53.8% in the current study aligns with global estimates ranging from 20–85% but is higher than the commonly reported average of 30% (5). This high prevalence may be attributed to the large proportion of severe strokes (53.3%) and limited access to early rehabilitation in this low-resource setting, a challenge common in LMICs (6). Additionally, the high prevalence of USN observed in the acute phase may have been partly influenced by unrecognized post-stroke delirium, which is common in acute stroke and can exacerbate attentional deficits, indicating the importance of comprehensive neurocognitive assessment in early stroke care (21).

The observed higher prevalence in the acute phase (60.4%) compared to the subacute phase (39.6%) suggests that some recovery occurs over time. Early disruptions of fronto-parietal attention networks, cerebral edema, diaschisis, and interhemispheric imbalance may contribute to transient but severe attentional deficits in the acute phase (10). These acute neurophysiological changes lead to transient but severe attentional deficits, which often improve as edema resolves, connectivity is restored, and neuroplastic reorganization occurs during the subacute and chronic phases. This temporal pattern is consistent with findings that USN severity often decreases with time, though persistent neglect can remain a barrier to recovery in some patients (11). To address the high USN prevalence in low-resource settings such as MRRH, there is a need to implement low-cost screening tools, such as paper-based line bisection or star cancellation tasks, and training community health workers and caregivers in visual scanning exercises that can facilitate early detection and management, to improve functional outcomes. Our findings further highlight the need for targeted rehabilitation for patients with severe stroke, strengthened acute stroke care, and timely initiation of secondary prevention and rehabilitation interventions.

Severe stroke was associated with a higher likelihood of USN, consistent with studies linking larger lesions to greater disruption of attention networks (12). The underlying mechanism involves damage to the fronto-parietal networks essential for spatial attention, which are often more severely affected in larger strokes (13). This association requires the need for prioritized screening and intensive rehabilitation for patients with severe stroke in similar low-resource settings, as their elevated risk of USN can lead to prolonged recovery, increased dependency, and higher healthcare costs(12). It also highlights the importance of strengthening acute stroke care, preventing secondary complications, and ensuring timely initiation of rehabilitation.

Right-hemisphere lesions were associated with a higher likelihood of USN, supporting the right hemisphere’s dominant role in spatial attention (5). This hemispheric asymmetry is well-documented, with right-hemisphere strokes more frequently causing neglect due to the brain’s lateralized attention systems (14). While the prevalence of USN was higher in right-hemisphere strokes, 40% of patients with left-hemisphere strokes also exhibited neglect, indicating that USN is not exclusive to right-hemisphere lesions. Therefore, while patients with right-hemisphere strokes may be prioritized for screening and early intervention due to their elevated risk, it remains important to assess all patients, including those with left-hemisphere strokes, to avoid missing individuals who could benefit from rehabilitation.

Similarly older age (≥60 years) was associated with a 1.66 times higher prevalence, likely due to reduced brain plasticity and vascular aging, which impair compensatory mechanisms (15,16). However, 43% of patients younger than 60 years also had USN, indicating the need for routine screening across all age groups. Prioritization may focus on older patients due to higher risk, but younger patients should not be excluded from assessment and early intervention programs. Right-handedness was associated with a lower likelihood of experiencing USN, possibly due to left-hemisphere compensation, though its clinical significance may be limited (17). Based on these findings, patients with left-handedness may warrant particular consideration for USN assessment. At MRRH, prioritization strategies for USN screening and intervention could focus on individuals with severe stroke, right-hemisphere lesions, and advanced age; however, universal screening remains important to ensure that younger patients and those with left-hemisphere strokes are not overlooked.

Overall, the high prevalence of USN in this setting indicates a gap in stroke care at MRRH, where routine screening for USN is not standard practice. Early detection and intervention, such as visual scanning training and prism adaptation, could mitigate USN’s impact on functional recovery (12,18). In LMIC settings like Uganda, where rehabilitation resources are limited, integrating low-cost, community-based interventions such as training caregivers in neglect management could be a feasible strategy (19). Additionally, targeting older patients and those with severe strokes through tailored rehabilitation programs could help address this burden (20).

## Limitations

SNAP primarily assesses visuomotor neglect, potentially missing auditory or other sensory deficits. In addition, SNAP relies on basic cognitive and language abilities; therefore, patients with conditions such as alexia or Wernicke’s aphasia, although not excluded, may have had difficulty completing the assessment, potentially confounding results. A small number of participants (n=3) were excluded due to inability to complete the assessment, which may reflect challenges related to attention, comprehension, or cooperation. These factors may partly explain the high prevalence of USN observed in this study population.

The cross-sectional design limits the ability to assess USN recovery trajectories, and the single-center study setting may limit generalizability to other healthcare contexts outside southwestern Uganda. Furthermore, we did not collect data on cardiometabolic risk factors, including diabetes mellitus, dyslipidemia, or cigarette smoking. The absence of these variables may have introduced residual confounding in our analyses, as they could influence stroke severity or outcomes, including USN. Delirium was also not formally assessed, particularly in the acute phase, which may have affected attention and task performance, potentially influencing the prevalence of USN observed. Future studies could incorporate multimodal neglect assessments, such as the Catherine Bergego Scale or Kessler Foundation Neglect Assessment Process (KF-NAP), include detailed cardiometabolic risk factors, and implement standardized delirium screening. Longitudinal study designs would also help capture the evolution of USN, particularly in patients with cognitive or language impairments, and improve understanding of functional outcomes in low-resource settings.

## Conclusion

This study revealed a high prevalence of USN among patients with stroke at a referral hospital in southwestern Uganda, affecting nearly half of the surveyed participants. Significant associations were found between USN and factors such as stroke severity, right-hemisphere lesions, advanced age, and right-handedness. The higher prevalence of USN in the acute phase highlights the importance of early detection and intervention. Targeted rehabilitation and early screening for patients with stroke with risk factors, particularly those with severe strokes or advanced age, could optimize recovery and improve long-term outcomes in this low-resource setting.

## Figures and Tables

**Table 1.** Characteristics of study participants, by unilateral spatial neglect status (N=117)

Variable	Overall (N=117) n (%)	USN Yes (n=63) n (%)	USN No (n=54) n (%)	P-value
**Age (Years)** ⁋				
<60	46 (39.3)	20 (31.7)	26 (48.2)	0.001
≥60	71 (60.7)	43 (68.3)	28 (51.8)	
**Sex**				0.568
Female	77 (65.8)	42 (66.7)	35 (64.8)	
Male	40 (34.2)	21 (33.3)	19 (35.2)	
**Education Level**				0.103
None/Primary	94 (80.2)	52 (82.5)	42 (77.8)	
Secondary/Tertiary	23 (19.8)	11 (17.5)	12 (22.2)	
**Employment Status**				0.008
Employed	38 (32.5)	19 (30.2)	19 (35.2)	
Not Employed	79 (67.5)	44 (69.8)	35 (64.8)	
**Stroke Type**				0.466
Ischemic	84 (71.8)	43 (68.3)	41 (75.9)	
Hemorrhagic	33 (28.2)	20 (31.7)	13 (24.1)	
**Stroke Severity (NIHSS)**				0.015
Mild (0–4)	20 (17.1)	7 (11.1)	13 (24.1)	
Moderate (5–15)	77 (65.8)	39 (61.9)	38 (70.4)	
Severe (16–42)	20 (17.1)	17 (27.0)	3 (5.5)	
**Lesion Location**				0.003
Right Hemisphere	63 (53.8)	41 (65.1)	22 (40.7)	
Left Hemisphere	54 (46.2)	22 (34.9)	32 (59.3)	
**Stroke Phase**				0.830
Acute (≤7 days)	71 (61.2)	38 (60.4)	33 (62.3)	
Subacute (>7 days to <6 months)	45 (38.7)	25 (39.6)	20 (37.7)	
**Handedness**				0.121
Right	105 (89.7)	51 (81.0)	54 (100.0)	
Left	12 (10.3)	12 (19.0)	0 (0.0)	
**Pre-Stroke mRS**				0.018
Independent (0–2)	107 (91.4)	55 (87.3)	52 (96.3)	
Dependent (3–5)	10 (8.6)	8 (12.7)	2 (3.7)	
**Hypertension History**				0.044
No	65 (55.6)	31 (49.2)	34 (63.0)	
Yes	52 (44.4)	32 (50.8)	20 (37.0)	

⁋ Median age=68 (IQR: 56–78) years; USN: Unilateral spatial neglect; mRS: modified Rankin Scale; NIHSS: National Institutes of Health Stroke Scale

**Table 2.** Factors associated with unilateral spatial neglect among patients with stroke at Mbarara Regional Referral Hospital, Uganda, August-December 2024

Variable	cPR	95% CI	p-value	aPR	95% CI	p-value
**Age (Years)**						
<60	Ref	-	-	Ref	-	-
≥60	1.82	(1.05–3.16)	0.033	1.66	(1.02–2.72)	**0.042**
**Sex**						
Female	Ref	-	-	Ref	-	-
Male	0.95	(0.58–1.56)	0.831	0.97	(0.61–1.54)	0.892
**Stroke Type**						
Ischemic	Ref	-	-	Ref	-	-
Hemorrhagic	1.27	(0.76–2.12)	0.392	1.15	(0.70–1.89)	0.584
**Stroke Severity (NIHSS)**						
Mild	Ref	-	-	Ref	-	-
Moderate	1.31	(0.67–2.55)	0.425	1.25	(0.65–2.41)	0.503
Severe	2.12	(1.08–4.17)	0.029	1.47	(1.12–1.92)	**0.005**
**Lesion Location**						
Left Hemisphere	Ref	-	-	Ref	-	-
Right Hemisphere	1.89	(1.18–3.03)	0.008	1.58	(1.14–2.19)	**0.006**
**Handedness**						
Left	Ref	-	-	Ref	-	-
Right	0.62	(0.41–0.94)	0.023	0.94	(0.94–0.99)	**<0.001**
**Pre-Stroke mRS**						
Independent (0–2)	Ref	-	-	Ref	-	-
Dependent (3–5)	1.65	(0.98–2.77)	0.059	1.19	(0.98–1.45)	0.070
**Hypertension History**						
No	Ref	-	-	Ref	-	-
Yes	1.45	(0.90–2.34)	0.126	1.42	(0.85–2.37)	0.176

cPR: crude prevalence ratio; aPR: adjusted prevalence ratio; USN: Unilateral spatial neglect; mRS: modified Rankin Scale; CI: Confidence interval

## Data Availability

The datasets used and/or analyzed during this study are available from the corresponding author on reasonable request.

## List of Abbreviations

aPR: Adjusted Prevalence Ratio
CI: Confidence Interval
LMIC: Low- and Middle-Income Countries
mRS: Modified Rankin Scale
MRRH: Mbarara Regional Referral Hospital
NIHSS: National Institutes of Health Stroke Scale
SNAP: Sunnybrook Neglect Assessment Procedure
USN: Unilateral Spatial Neglect
